# President’s Address before the Alumni Association of the Southern Medical College*Delivered July 20, 1898, at the meeting held at Atlanta, Ga.

**Published:** 1898-09

**Authors:** J. McF. Gaston

**Affiliations:** Atlanta, Ga.


### PRESIDENT'S ADDRESS BEFORE THE ALUMNI ASSOCIATION OF THE SOUTHERN MEDICAL COLLEGE.*

BY J. McF. GASTON, JR., A. M , M. D., ATLANTA, GA.

The Southern Medical College was opened October 13, 1879, when Dr. T. S. Powell, professor of obstetrics in the college and founder, delivered the opening address. He took for his subject the Bible sentence, "Whatsoever a man soweth, that shall he also reap," and he made a strong presentation of the claims of the new institution on the grounds of honor, ethics, high standard of teaching, and laudable ambition to excell. He denied the insinuations of enemies, gently rebuked the pessimists, and at the same time realized that the optimists are not always right. He preferred to rely on his sense of the encouragement which the future would bestow upon his enterprise and that of so many earnest, noble co-operating spirits. He trusted in God, and was impelled by a desire to be classed among the benefactors of mankind, such as he deemed Howard Payne and all the founders of Yale, Harvard and other educational institutions. He had no words of bitterness, but believed in the motto of "Live and let live."

A few well-worded sentences out of many gems of thought may be here quoted with great profit. "The object and the earnest desire of the founders of the Southern Medical College of Atlanta is to legitimately and thoroughly teach the science and the practice of medicine upon a broad, high and progressive basis. No man should have and does have more of the spirit of progressive knowledge than the true physician, but to elevate the profession to the desired eminence he must have the sympathy and support of the public, its confidence in his efforts, and its respect for them."

The child who when asked where home was, said, "Where my loved ones are," was not confined in area to four walls or to any street number, or to the few men arrogating the power to collect rent. The alumni will never regard the Southern Medical College changed in name or in honor, or in deed, until the love of the graduates shall be transferred to another *alma mater*.

In connection with the love of home, of home institutions, and of the Southern Medical College, allow me to quote again from Dr. Powell's introductory address:

"'The Home, Sweet Home.' of John Howard Payne is a stereotyped melody around the fireside in every land where the song has been sung, and the beautiful self-immolated life of his illustrious namesake is a precious legacy to the whole philanthropic world.

"In our own country the time-honored names of Yale and Harvard are not enshrined only in the memory of their bene-

*Delivered July 20, 1898, at the meeting held at Atlanta, Ga.

ficaries among our Northern friends, but North, East, South and West have paid their tribute of eulogy to these halls of learning, and in every section of the Union there are found alumni of these institutions, who to the close of their lives will have vivid and grateful recollections of their loved and revered alma mater.

"The venerable William and Mary and Randolph-Macon Colleges, though erected on Southern soil, open their fountains of knowledge to every one, irrespective of clime or nationality, who would drink of the Pierian Spring, and the more modern institution, Washington and Lee College, has already a world-wide fame, wherever the names of these our illustrious chieftains are known and honored."

"What has been reaped?" may be asked me as one who dares to write a history of the institution known as the Southern Medical College. I answer, that among the many things reaped may be enumerated a three-years' graded course of lectures, a well recognized position among the examining boards of all the States, but especially Tennessee, Alabama,* Georgia, and Virginia.† The address of Dr. B. J. Baldwin, as president of the Alabama Medical Association, and the many references by others to the Southern Medical College are facts. The Southern Medical College had twenty applicants for license to practice before the examining board of Alabama, and the record gives seventeen granted and three rejected. It is well, to bear in mind in this connection that the Southern Medical College was then several years younger than it is to-day, that the applicants had only been through two courses of lectures, and that there was no selection whatever of the material examined. The fact that the Atlanta Medical College had over double (forty-two) to apply and yet only twenty-eight, seven over one-half of this number, were granted, shows the marked disproportion, which is still further emphasized by the comparison of fourteen rejected to our three. Where a natural proportion would make the number at most six, it is over double what it should show when compared with the record of the younger school. But how did Augusta compare as the seat of the medical department of the University of Georgia? Eight applied, six granted, two rejected. Increase by multiplying by five all the numbers, and we approach near the number who applied in the Atlanta, forty, and we have thirty to compare with twenty-eight who were granted, and ten with fourteen who were rejected.

---

*Southern, per cent, 15–85; Augusta, per cent., 25–75; Atlanta, per cent., $33\frac{1}{3}$–$66\frac{2}{3}$; Eclectic per cent. 40–60. The first numbers represent licenses rejected, the second granted, from 1878, to 1892.

†We find an official statement, or table, in the Virginia Medical Semi-Monthly of August 12th 1898. The Medical Examining Board of Virginia from the organization of the Board, January 1st, 1885 to June 24, 1898—has had four applicants from the Southern Medical College, aud the number licensed on first examination was two, while two were rejected. The Atlanta Medical College had only two to apply, but both were rejected.

How about the two other Georgia colleges mentioned in this list? The Savannah Medical College had one to apply, and that was granted. This college, by the way, has been allowed to stop its good record by suspending operations. But as to the Georgia Eclectic College of Medicine and Surgery we have the figures thus: Ten applied, six granted, four rejected. Double, and we have the first number for comparison with the Southern, and we have twelve granted to our seventeen, and eight refused to our three. And yet the Georgia College of Eclectic Medicine and Surgery is able to stand alone, and the Southern Medical College has been represented by some as needing support.

During the transition stage between the second and third courses of lectures, one of our present alumni, Dr. A. Browne Evans, of Churview, Va., wished to practice and was licensed to do so by passing the examination of the examiners of the State of Virginia.

From a patronage chiefly confined to our own State the Southern Medical College began to draw students from all States, and in the last year's list of matriculates may be found men from Wisconsin and Cuba. But it it was distinctly what its name implied, and that which we shall always cherish —the *Southern* Medical College.

In the same number of the SOUTHERN MEDICAL RECORD in which we find Dr. Powell's eloquent address upon "Southern Institutions," is the statement from Dr. Word, the first dean, that the college had fifty matriculates. "An extraordinary opening, truly," he remarks, for a new institution. Again we find with the initials T. S. P. a strong editorial on the "Green-Eyed Phantom," jealousy.

It also appears that quite a number of valuable books were donated by D. Appleton & Co. to the Southern Medical College library.

As supplementary matter I here incorporate by permission information which has been communicated to me by Dr. W. T. Goldsmith, in a private interview. Dr. Goldsmith himself has been connected with the Southern Medical College since the organization. He was one of the first professors and trustees. He was co-editor of the SOUTHERN MEDICAL RECORD with Dr. Powell and Dr. Word. He states that, on ethical grounds, Dr. Powell had been opposed by many of his colleagues who were not willing to allow of an honest difference of opinion and packed the Medical Association of Georgia in order to secure their vindication. Dr. Duncan, of Savannah, wrote to Dr. Goldsmith, advising that Dr. Powell and he move in the matter of erecting a new college, promising the support of their Savannah colleagues. Dr. Powell was shown the letter and began to organize what became the Southern Medical College.

While the North is overcrowded with medical schools, the number of students yearly who go from the South to Northern schools testifies to the necessity for a higher course of study in the medical colleges in the South. Four years and no less should be the graded course of every college in the South, and the recent resolution passed by the American Medical College Association setting as the limit for the admission of members of colleges having only three years' course to the American Medical Association the year 1899, will, we hope, compel the members of the faculties of all institutions in the South to adopt a four-years' graded course.

It may be said that the University of New York and Bellevue consolidated, that Niagara and the University of Buffalo consolidated, but it will always be understood that the Southern Medical College was bought or absorbed by the Atlanta Medical College; and, far from a higher standard, we expect to see a lower standard of education. But the history of the Bellevue Hospital Medical College* shows that Cornell may claim quite as much of the benefit of a graft from it as the University of New York, while the *Buffalo Medical Journal* acknowledges that Niagara was the weaker vessel.

The Southern Medical College has had a varied and rugged road to travel. Starting with nothing but the indomitable will of Dr. T. S. Powell and the hearty co-operation of an able faculty, it seemed to be made for better things; and the motto was thorough instruction in medicine, surgery, and allied sciences. The graduates were gradually becoming a class of cultured and scientific physicians, and no man dared to impugn their honesty and devotion to right and duty.

The older men, seeing the advances of the last decade, have fitted themselves by study abroad or by the attendance upon post-graduate institutions or medical societies. The younger men, having derived great benefit from the increased facilities for study and work in the laboratory and the hospitals, have taken a stand with the foremost. In verification of these remarks we point to the rolls of the various State medical societies, to their county and city societies, and, especially, to their hospitals, beginning with Atlanta, Macon, and ending in the Indian Territory, Colorado, and California.

But the strength of any college is the devotion of the alumni, and I have yet to see a man among her alumni who has failed to revere and to hold in great and unbounded respect the memory of the Southern Medical College. But what can we do? We can act as a nucleus for mutual improvement, and who knows the influence and the service to our former teachings in ethics and on medicine and surgery we may be.

*Cornell University Medical College has four professors formerly in Bellevue, while the University and Bellevue Hospital Medical College has thirty-three professors and assistants formerly connected with the faculty of Bellevue Hospital Medical College, but Cornell has eleven formerly in the University of New York while the University and Bellevue has only five professorships of importance from among the number on the staff of instruction as given in the annual announcement of the University of New York for 1894-95.

The day we meet is the day for the reunion of the Confederate Veterans. Do these veterans regard the Southern cause they fought for any less dear to their hearts because the Confederate States are now confederated with the United States? No, never. They are separate and distinct, and we meet today to honor the Confederate Veterans for their work. And we, of the Alumni Association of the Southern Medical College, are here to represent the old and not the new college.

"The old oaken bucket, the iron-bound bucket, that hung in the well," was far more fit to give drink to our poet than the most costly substitute could have been, even if it had been made of gold and had been studded with diamonds.

The men from whom we received our own medical lore have been called to rest, or are "fast passing away and the places that once knew them, shall know them no more." The teachings of such men as Drs. Word, Powell, W. D. Bizzell, Thad Johnson and others, however, linger and bear fruit at the bedside.

"The age of a man is purely relative" is a sentence I heard from Dr. Bizzell before I entered college. Although I never heard him in college hall, yet I rejoice to know that I did have the benefit of a close friendship, and that these words were uttered in a Christian hall, and embellished a part of his conception of the ideal man throughout the ages.

I never stay long beside an obstetric case that I do not recall the teachings of Dr. T. S. Powell. I did not have the pleasure of hearing or personally knowing Dr. Thad Johnson, but I hold his memory in deep esteem because of the fact that he he preceded another whose connection with the faculty of the Southern Medical ceased by voluntary resignation only a day or two before the opening wedge for a so-called consolidation was driven with soft and easy licks into the very vitals of the old Southern Medical College. Yet I knew Dr. Word well, and most heartily did I appreciate his high and lofty spirit. Of the successors of these men, no word from me need be said, and I leave the living to live on until the true historian may do them justice. My college days were pleasant days, and I there formed some very lasting friendships.

To my own unaided view, clouds here and there could be seen, and yet, while I apprehended the worst, I hoped for the best. The spirit of the demagogue crept in occasionally, but over all was a controlling and pacifying element which tended to abase self, and to instill unselfish devotion to duty and to the interests of the Southern Medical College. The good is not always aggressive, while the bad is ever multiplying. While the present is not an effort at a funeral of the Southern Medical College, still I will quote the words of Mark Antony uttered at the funeral of the great Cæsar:

"The evil that men do lives after them;
The good is oft interred with their bones."

But even as there is a resurrection of the body after death, so there is a resurrection of a college.* "Principles live" and need exponents. The alumni of the Southern Medical College are her exponents.

The old aphorism runs, "Truth crushed to earth will rise again," while the same idea may be expressed in this way that other cities and other colleges will teach the principles needed, if our own colleges and our own State refuse them.

Let us hope for the best. In the words of Daniel Webster, "May that fear of God which expels all other fear, and that regard for duty which transcends all other regard," influence our doctors, and may the fact that a man seeks an office or plots for another man's office be sufficient reason for suspecting his own good faith and his professions of loyalty to any cause. "An office should be neither sought nor declined."

[*NIAGARA MEDICAL COLLEGE.—A short time ago Niagara Medical College, of Buffalo, consolidated with the University of Buffalo. Before all the books and laboratory appliances had been removed from the Niagara Medical School the rejected faculty got together, reorganized, and are preparing to reopen the Niagara Medical College.—*Philadelphia Medical Journal.*]

N. B.—In the announcement of the medical department of Cornell University which has been issued since this address was delivered and came in the mails September 8, 1898, great stress is laid npon the fact that the members of the faculty of the medical department of another university had been secured. All will recognize in the names of Wm. M. Polk, dean, Lewis A. Stimson, C. S. Bull, F. S. Dennis, F. Kammerer, R. A. Witthaus, Austin Flint, and others, a galaxy of names unsurpassed in the annals of the medical education of the last quarter of a century. The increased attention bestowed upon the system of recitations from standard text-books and the bedside instruction augurs well for its future influence on our doctors. If the consolidation of Bellevue Hospital Medical College has caused the great institution at Ithaca, New York, to found a medical department in the city of New York, we may expect the former teachings of Bellevue Hospital Medical College to be continued in tact rather than to be absorbed, while the Medical College of the New York University continues. The present high standing of Cornell University in the academic department at Ithaca, where the first two years of the medical student are allowed, without, however, preventing a change of the regular full four years' course in New York City, should be helpful. More medical colleges are thus being the result of an effort at consolidation. More professors are being brought to the centres of medical teaching, and more buildings are being erected.

The effect is also in decreasing the number of pupils in attendance in one institution rather than in consolidating the students, so that we have laboratories and hospitals, where sections of students may be prepared for their life-work.

CORNELL UNIVERSITY MEDICAL COLLEGE has received from Col. Oliver H. Payne the gift of one and one-half million dollars, and work is to be at once begun on the new building, five stories high, at First avenue and Twenty-seventh street, New York, which it is proposed to build and endow. Colonel Payne, it will be remembered, formerly gave $150,000, which, finally, after much discussion, benefited the medical department of the University of New York. The present magnificent gift will at once place the Cornell University Medical Department among the foremost of the country. The course will require four years, the first two of which may be taken at Ithaca. While heretofore money has been found for every other public institution, it has been strange how little benefactors have recognized the need of it for health, the most important of all things. The public ingratitude to the medical profession and indifference to its own physical well-being are the strangest of psychologic mysteries. It may be hoped that this incomprehensible stupidity is at last to end. The scandals of State appropriations and medical politics are becoming insupportable, even if there were no higher motive.—*Philadelphia Medical Journal, September 17, 1898.*

[We concur in this hope.]

---

### KOPLIK'S NEW SIGNOF MEASLES.

Slawyk (*Deut. Med. Wochenschr.*, April 28, 1898) is astonished that no one should have noticed until recently the early eruption which occurs on the mucous membrane of the lips and cheeks in measles, as described by Koplik of New York, and which is said to be absolutely characteristic of the disease. The sign is of especial importance in asylums and hospitals for children where, as is well known, the mortality in epidemics of measle is far higher than is seen in private practice, often reachingthirty per cent. or higher, and where children not unfrequently suffer from repeated attacks of the disease.

